# The attitudes of typically developing adolescents towards their sibling with autism spectrum disorder

**DOI:** 10.4102/sajcd.v64i1.184

**Published:** 2017-04-26

**Authors:** Christine van der Merwe, Juan Bornman, Dana Donohue, Michal Harty

**Affiliations:** 1 Centre for Augmentative and Alternative Communication, University of Pretoria, South Africa; 2 Department of Psychological Sciences, Northern Arizona University, United States; 3 Division of Communication Sciences and Disorders, Department of Health and Rehab Sciences, University of Cape Town, South Africa

## Abstract

**Background:**

Understanding how the cognitive, emotional and behavioural components of sibling attitudes interact with one another at various stages of a sibling’s lifespan will allow clinicians to provide better support to children with autism spectrum disorder (ASD) and their families. However, no research exists which focusses on describing the attitudes of adolescent siblings of children with ASD within the South African context towards their sibling with an ASD. The primary aim of this study was to investigate how typically developing adolescents recall their past attitudes and describe their present attitudes towards their sibling with an ASD.

**Methods:**

Thirty typically developing adolescents who have siblings with ASD were selected to complete the survey instrument, the Lifespan Sibling Relationship Scale, using a cross-sectional design.

**Results:**

Results indicate that the measure has internal consistency within this sample. Wilcoxon signed-ranks tests were used to test for significant differences between the mean values for the two self-reported time periods. Friedman analysis of variances (ANOVAs) was used to test for significant differences in the three components of attitudes, namely affect, behaviour and cognition. Results indicate that participants held more positive attitudes towards their siblings with ASD as adolescents compared with when they were younger and that adolescents rated their current emotions towards and beliefs about their sibling with ASD to be more positive than their current interaction experiences.

**Conclusion:**

As siblings’ attitudes appear to change over time, clinicians should use a lifespan approach to sibling attitudes when designing and implementing supports for siblings of children with ASD.

## Introduction and aim

Similar to international trends, the prevalence of autism spectrum disorder (ASD) in South Africa appears to be increasing (Bateman, 2013). A recent South African study which investigated ASD rates between 1996 and 2005 reported an 8.2% increase in the number of children with features of ASD at a clinic in the Western Cape during this period (Springer, van Toorn, Laughton & Kidd, 2013). ASD is characterised by persistent deficits in the areas of social interaction, language and communication, behaviour and thinking, and sensory processing (American Psychiatric Association, 2013). These difficulties impact the way that individuals with ASD develop, understand and maintain social relationships with others. Characteristics of ASD that make social interaction difficult include the lack of joint attention and turn-taking (Prelock & Nelson, 2012), limited use of gesture (Ellawadi & Ellis Weismer, 2014) and limited age-appropriate spoken language (Ellis Weismer, Lord & Esler, 2010). The early social attention difficulties associated with ASD often lead to cascading effects on children’s learning and social relationships (Adamson, Romski & Barton-Hulsey, 2014).

Sibling interactions often provide children with ASD with their first socialisation experiences (Meadan, Stoner & Angell, 2010). During childhood, children will spend more time with their siblings than with any other individual (Orsmond & Seltzer, 2007). Therefore, it is not surprising that the role of siblings (especially older ones) on speech, language and communication development has been considered in the literature (Marshall, Goldbart & Phillips, 2007).

The aforementioned core characteristics of autism (*vis á vis* difficulty with social communication, maintaining eye contact, the presence of restrictive and repetitive behaviours, and sensory processing difficulties) make it more challenging for typically developing children to interact with their sibling with ASD (Ferraioli, Hansford & Harris, 2012). The increase in prevalence of ASD means that more children have siblings with ASD (Diener, Anderson, Wright & Dunn, 2014), and thus, it is important to understand the siblings’ psychosocial adjustment when their sister or brother has ASD.

The results of research regarding the impact of ASD on siblings’ psychosocial adjustment have been mixed (see Meadan et al., 2010 for a review), with some studies reporting negative influences of having a sibling with ASD (Bitsika, Sharpley & Mailli, 2015; Macks & Reeve, 2007; Meyer, Ingersoll & Hambrick, 2011) and other studies reporting positive influences (Kaminsky & Dewey, 2001). Interestingly, there are also studies in which individuals acknowledge the presence of mixed emotions or emotional ambivalence towards their sibling with ASD (Angell, Meadan & Stoner, 2012; Petalas, Hastings, Nash, Lloyd & Dowey, 2009). Angell et al. (2012) report that typically developing siblings related examples of typical sibling experiences, as well as negative experiences of embarrassment related to the challenging behaviour demonstrated by their sibling with ASD. Petalas et al. (2009) suggest that, at least for some siblings, a tension exists between accepting their sibling with ASD while still wanting aspects of the ASD to be alleviated. They (Petalas et al., 2009) state:

It is not clear whether the children actively held these tensions between acceptance and change, or whether these often contradictory views were evidence of ongoing cognitive processing and appraisal. (p. 389)

Notwithstanding this lack of consensus in the literature, it is clear that typically developing siblings of children with ASD hold varying degrees of positivity towards their relationship with their sibling with ASD.

On the contrary, a recent systematic review that investigated the effectiveness of sibling support for children with intellectual disabilities indicates that not all siblings are likely to be in need of support (Tudor & Lerner, 2015). Tudor and Lerner (2015) indicate that factors such as demographic status and degree of challenging behaviour exhibited by the sibling with intellectual disability, as well as gender and birth order of siblings, may influence sibling adjustment. Although not specific to ASD, this systematic review highlights the need for more data to be collected, which will assist in determining which subgroup of siblings may benefit from more intensive sibling support. In addition to the demographic and familial differences that impact on the need for sibling support, siblings’ attitudes towards, and understanding of, the disorder is known to change over time. They have been linked to the cognitive skills a sibling can employ when assigning meaning to the experiences they share with the child with ASD (Glasberg, 2000; Riggio, 2000). Consequently, understanding how the cognitive, emotional and behavioural components of sibling attitudes interact with one another at various stages of a sibling’s lifespan will allow clinicians to provide better support for children with ASD and their families.

To capture the changes in the sibling relationship over time, Riggio (2000) developed the Lifespan Sibling Relationship Scale (LSRS). This scale measures three dimensions of the sibling relationship in both childhood and adulthood, namely positivity of behaviour, beliefs about the relationship and affect towards the sibling. This self-report scale differs from other scales that measure sibling relationships, in that it measures past and present attitudes towards the sibling with ASD. The development of the scale was based on the conceptualisation that attitudes comprise three different components, namely cognitive, behavioural and affective components. Oppenheim (2000) explains how these three components are interrelated and states that beliefs (the cognitive component) often elicit very strong feelings (affective component). Together, these result in specific actions being taken (behavioural component) (Oppenheim, 2000).

It is, therefore, evident that the sibling relationship between typically developing siblings and children with intellectual disabilities, including autism, has received substantial attention in international literature. However, there are only a handful of South African studies that explore issues relating to siblings of children with developmental disorders or disabilities. The authors are aware of two studies that have been conducted that explore the attitudes of siblings of children with severe speech and language difficulties (Hansen, Harty & Bornman, 2016), and the attitudes of siblings of children with severe disabilities (Opperman & Alant, 2003). Hansen et al. (2016) indicated that older siblings were most positive towards completing household tasks and playing with their younger sibling with speech and language difficulties, less positive about their ability to communicate with their younger siblings and the least positive about the effect that having a younger sibling has on their own ability to build and maintain relationships with family members, such as parents. Opperman and Alant (2003) found that typically developing siblings felt inadequately supported and excluded from the intervention process, which resulted in atypical family participation and interaction patterns. Together, the results of these studies suggest that while typically developing siblings enjoy interacting with their sibling with a disability, they require additional information and support to navigate the more challenging aspects of the sibling relationship.

To date, however, no research exists which focusses on describing the attitudes of adolescent siblings of children with ASD within the South African context towards their sibling with ASD. This study, therefore, sought to describe and compare participants’ self-reported past and present attitudes towards their sibling with ASD, as well as to explore the association between the components of attitudes and the two life stages under examination, namely childhood and adolescence.

## Material and methods

### Design and measures

This study uses a cross-sectional survey design. Given that both of these attitudes (past and present) were assessed at a single point in time, it is partially a retrospective design.

The LSRS (Riggio, 2000) was used to collect the data for this study. This tool is a self-report survey and differs from other attitudinal scales as it captures self-reported attitudes of adults as well as their memory of attitudes they held as children. The scale has been developed to measure three dimensions of the sibling relationship (affect, behaviour and cognition), and captures these dimensions in childhood and adulthood (Riggio, 2000). The scale comprises 48 items divided into six subscales. Child affect (CAff) and adult affect (AAff) measure emotions towards the sibling. Child cognition (CCog) and adult cognition (ACog) assess beliefs about their sibling and their sibling relationship in the respective developmental phases. The measurement of how positive behavioural interactions were, or are, comprises child behaviour (CBeh) and adult behaviour (ABeh). The scale is, thus, unique in that it captures current views as well as requests the participant to recall earlier views they held as a child. The items are answered on a five-point Likert Scale, with ‘1’ indicating strong disagreement with the statement and ‘5’ indicating strong agreement. As the participants in this study were adolescents, the ‘A’ in the scale names will refer to adolescent instead of adult as in the original scale, while the ‘C’ for child remains unchanged, that is, child and adolescent affect (CAff and AAff), child and adolescent behaviours (CBeh and ABeh) and child and adolescent cognition (CCog and ACog).

The psychometric properties of the LSRS have been established with a sample of 711 undergraduate and graduate students (62% female and 38% male). The LSRS (Riggio, 2000) has been validated and demonstrated high internal consistency for all six subscales (with a range from α = 0.84 to α = 0.91) and the instrument as a whole (α = 0.96). There is also evidence of a coherent factor structure and stability of responses over time (Riggio, 2000). For the purposes of this study, we made a minor modification to the wording of the scale. In the questions on the three subscales dealing with attitudes held as children, the phrase ‘as a child’ was replaced with ‘when we were younger’, as the siblings were adolescents, not adults. Sample questions from each subscale and the original subscale α-values can be viewed in Table 1. Key biographical information was also collected using a questionnaire, specifically designed to capture information relating to participants age and gender, number of siblings in the house, and the age and gender of siblings. This questionnaire was completed at the same time as the LSRS.

**TABLE 1:** Sample items from the Lifespan Sibling Relationship Scale modified for completion by the adolescents.

Variable	Attitude components	Statement	Riggio (2000) α-value†
Child	Affect	I remember loving my sibling a lot when we were younger.	0.89
	Behaviour	My sibling and I spent time together after school when we were younger.	0.84
	Cognition	My sibling and I had a lot in common when we were younger.	0.88
Adolescent	Affect	My sibling frequently makes me angry.	0.91
	Behaviour	My sibling and I do a lot of things together.	0.87
	Cognition	I believe that I am very important to my sibling.	0.91

† , total scale α = 0.96.

### Procedure

Permission to conduct this study was obtained from the relevant ethics authorities. Data were collected at two special schools for children with ASD, one in Cape Town and the other in Pretoria, as part of a ‘Sibling support day’. The aim of the day was to provide information to siblings about ASD, as well as to create an opportunity for them to discuss issues of concern in a safe environment. Content covered included the children’s understanding of what ASD was, the different expectations parents may have of the adolescents, compared with their sibling with ASD, the siblings’ experience of having a sibling with ASD (both the positive and negative aspects) and what their hopes and fears were regarding the sibling at that point in time as well as in the future. The LSRS was completed prior to the commencement of the activities for the Sibling support day, to ensure that the planned activities did not influence the adolescents’ responses. Both of the Sibling support days were facilitated by the first author.

A flyer was distributed through the networks of Autism Western Cape and Autism SA. Letters were also sent to schools specialising in the education of children with ASD in both Cape Town and Pretoria. Parents received a flyer containing information about the study, which they were encouraged to share with the participants. Written consent was obtained from the parents of the sibling. Before data were collected, the adolescents themselves also provided written assent. There were a number of families who indicated a willingness to participate but were unable to attend either of the Sibling support days for a variety of reasons. A copy of the survey was sent to these typically developing siblings either through email or through the school, depending on their personal preference. They completed the survey at home and returned it to the researcher either through email or through the school.

### Participants

Thirty typically developing siblings completed and returned the questionnaire. The demographics of the participants and their families were collected using a biographical questionnaire developed specifically for this study. Thirteen participants completed the questionnaire during one of the two Sibling support days, whereas seventeen (who wanted to complete the questionnaires but who could not attend the Sibling support days) completed the questionnaires at home. A Fischer’s exact test indicated that there was no significant difference between the group who completed their questionnaire at the Sibling support day and those who completed the questionnaire at home, in terms of gender (*p* = 0.9999) and number of siblings in the household (*p* = 0.2608). Since the two groups were functionally equivalent on these characteristics, the data from both groups were analysed together as one data set (*N* = 30).

The majority of the adolescents were living in the same house as their sibling at the time of data collection (90%, *n* = 27). Ninety per cent (*n* = 27) of the parents were married at the time of data collection. Twelve participants came from families with two children, 13 participants had three children in the family and five participants had four or more children in the family.

The ages of the adolescent siblings ranged from 13 to 20 years (M = 15 years; SD = 1.69). An equal number of male participants (*n* = 15) and female participants (*n* = 15) completed the survey. The majority of the typically developing adolescents (*n* = 27) were older than their sibling with an ASD.

The age of the siblings with ASD ranged from 7 to 21 years (M = 10 years; SD = 3.50). Ninety per cent (*n* = 27) of the children with ASD were male and all had received a formal diagnosis of ASD, which would have been confirmed at the time in which they enrolled for formal schooling (using the Diagnostic and Statistical Manual of Mental Disorders, 4th Edition (DSM IV) criteria). The children all attended a special school for children with ASD (either in Cape Town or Pretoria) and had access to the necessary therapeutic support through the school.

### Data analysis

Data were analysed using the Statistical Package for the Social Sciences 24 (SPSS). Firstly, data were checked for accuracy, normality and outliers. The Shapiro–Wilk test for normality suggested that each of the subscales exhibited normality (AAff: *p* = 0.08, ABeh: *p* = 0.72, ACog: *p* = 0.44, CAff: *p* = 0.11, CBeh: *p* = 0.26, with the exception of CCog: *p* = 0.02). Because subscale CCog violated the assumption of normality, non-parametric analyses were used to test for significant differences between the mean values for the two self-reported time periods (‘now’ and ‘when we were younger’). Friedman two-way analysis of variance (ANOVA) was used to test for significant differences in the components (affect, behaviour and cognition) of attitudes between the two self-reported time periods.

### Internal consistency reliability in this study

In order to test whether the LSRS (Riggio, 2000) retained its internal consistency with the modifications which were made for the purposes of the study (the cognition subscale was reduced from eight items to seven items in this data set due to missing data on the last CCog item). Cronbach’s alpha values were obtained for each of the six subscales. The Cronbach’s alpha values for each subscale ranged from 0.71 to 0.97 (total scale α = 0.83), which indicated a satisfactory level of internal consistency. These values can be viewed in Table 2.

**TABLE 2:** Means, standard deviations and Cronbach’s alpha values for the three subscales and Wilcoxon rank mean difference and *z*-scores comparing attitudinal differences in childhood and adolescence (*N* = 30).

Components of attitude	Adolescent			Child			Difference	
		
	Mean	SD	Cronbach’s alpha†	Mean	SD	Cronbach’s alpha	Mean rank diff	*z*-score
Affect	3.80	0.75	0.92	3.38	0.89	0.97	11.45	-4.25*
Behaviour	2.62	0.69	0.80	2.79	0.64	0.79	0.48	-1.25
Cognition	3.65	0.79	0.86	2.81	0.62	0.71	6.36	-3.74*

† , total scale α = 0.83.

* , *p* < 0.01.

## Results

### Comparison of the attitude according to life stage

In order to compare the components of present attitudes towards siblings with ASD to past attitudes, Wilcoxon signed-rank tests were run on the data, which are also presented in Table 2.

The first analyses were conducted to compare participants’ scores between child and adolescent affect. The results showed a statistically significant increase from CAff (mean rank = 17.85) to AAff (mean rank = 6.40), *z* = -4.87, *p* < 0.01, *r* = 0.63, where participants rated their AAff scores significantly higher than their CAff scores. The second analyses compared participants’ scores for behaviour, CBeh (mean rank = 14.18) and ABeh (mean rank = 13.70), which indicated no significant difference *z* = -1.25, *p* > 0.05. The final analyses determined whether or not there were differences in participants’ scores on cognition and compared CCog with ACog. The results showed a statistically significant increase from CCog (mean rank = 16.56) to ACog (mean rank = 10.20), *z* = -3.74, *p* < 0.01, *r* = 0.48, where participants’ ACog scores were significantly higher than their CCog scores.

### Comparison of attitude components within each life stage

In order to gain a better understanding of the three components of attitudes, a Friedman ANOVA was conducted to explore how the components of attitudes (affect, behaviour and cognition) differed within each life stage.

The first ANOVA showed that there were significant differences within child attitudes, χ^2^ (2, *N* = 30) = 47.41, *p* < 0.01. To identify which attitudes differed, a series of Wilcoxon ranked sum tests with Bonferroni corrections were run (*p* < 0.016). The results showed a statistically significant decrease from CAff (mean rank = 2.88) to CBeh (mean rank = 1.71), *z* = -4.50, *p* < 0.01, *r* = 0.58; a statistically significant decrease from CAff (mean rank = 2.88) to CCog (mean rank = 1.41), *z* = -4.68, *p* < 0.01, *r* = 0.60; and a non-significant decrease from CBeh (mean rank = 1.71) to CCog (mean rank = 1.41), *z* = -1.80, *p* = 0.07. The second ANOVA showed that there were significant differences within adolescent attitudes, χ^2^ (2, *N* = 29) = 35.81, *p* < 0.01. The results showed a statistically significant decrease from AAff (mean rank = 3.00) to ABeh (mean rank = 1.32), *z* = -4.79, *p* < 0.01, *r* = 0.62; a statistically significant decrease from AAff (mean rank = 3.00) to ACog (mean rank = 1.68), *z* = -4.87, *p* < 0.01, *r* = 0.63; and a significant decrease from ACog (mean rank = 1.68) to ABeh (mean rank = 1.32), *z* = -2.42, *p* = 0.015, *r* = 0.31.

## Ethical consideration

All procedures performed in studies involving human participants were in accordance with the ethical standards of the institutional or national research committee and with the 1964 Helsinki Declaration and its later amendments or comparable ethical standards. Informed voluntary consent was obtained from all individual participants included in the study.

## Discussion

The purpose of this study was to examine and compare adolescents’ past and present attitudes towards their siblings with ASD. Attitudinal scores were calculated in the areas of affect, behaviour and cognition. There are two main findings from the study. The first is that the results of the analyses indicated that the adolescents who participated in this study experienced their relationships with their siblings with ASD more positively as they grew older. The second point to discuss is the significant differences found within the attitudinal components within each of the two life stage periods (childhood and adolescence).

A possible explanation as to why the adolescents in this study reported that their relationships became more positive as they grew older is that over time the adolescents’ abilities to understand and cope with their siblings with ASD may have increased. Research suggests that, compared with younger children, adolescents are more able to engage with material explaining autism and its effects (Bloch & Weinstein, 2009; Petalas et al., 2009). These participants may thus have developed the cognitive ability to better understand the characteristics of ASD and become more empathetic with their siblings. Accordingly, our findings are in agreement with the work of Glasberg (2000), who suggests that adolescents between the ages of 11 and 17 years are able to reason logically about the past as well as present events and that they are able to use past and present experiences to project the implications of their sibling’s disability on their sibling’s life, as well as their own. Thus, the higher level of cognitive thinking aligned with adolescence allows adolescents to develop more positive attitudes towards their siblings as they get older. Moreover, they may also have developed more appropriate coping strategies as they grew into adolescence.

A significant difference was found between the scores of the participants’ affect and behaviour components when compared with their cognition scores during adolescence. In other words, adolescents rated their present emotions towards their sibling with ASD (affect component) and their present beliefs about their relationship with their sibling with ASD (cognition component) higher than their present positive interactions with their sibling with ASD (behaviour component). This may be due to the fact that, no matter how positive their feelings towards their sibling are and how well they understand ASD, they still need to manage their own behaviour in relation to the obstacles that their sibling with ASD is likely to have in social relationships (Rivers & Stoneman, 2003). Larson (1998) refers to a similar concept, defined as the ‘paradox of disability’. On the one hand, the typically developing sibling loves the sibling with ASD as he or she is, while also wanting to erase the disability. If typically developing siblings could be taught to embrace the paradox of disability, they could attempt to create a positive bias that could fuel their optimism in the sibling relationship.

Participants were also asked to recall their past attitudes about their sibling with ASD when they were both younger. Within the child life stage, a significant difference was found between their higher affect scores when compared with their lower behaviour and cognition scores. Thus, when participants recalled their relationship with their sibling with ASD when they were younger, they reported that they felt more positive emotion towards their sibling with ASD (affect component) compared with their feelings about interactions with their sibling with ASD (behaviour component) and beliefs about their relationship (cognition component). Perhaps this is their own reflection of their limited awareness of how to navigate atypical interactions when they were younger. These data corroborate results from Bitsika et al. (2015), which suggests that sibling interventions may need to focus on some attitudinal areas more than others in order to foster the sibling relationship. As suggested by Bitsika et al. (2015), teaching siblings of children with ASD to control their emotions and so change their behavioural responses towards their sibling with an ASD can be an important intervention topic for support or sibling intervention to encourage positive social interactions between them.

However, a recent systematic review indicated that clinicians should be cautious in applying a one-size-fits-all approach to sibling support. Tudor and Lerner (2015) suggest:

In addition to psychological and behavioural functioning and sibling impairments, familial psychopathology, problematic family functioning, amount of attention given toward siblings, and sibling social skills impairments could all be of import in identifying at-risk youth and selecting appropriate services. (p. 18)

Acknowledging this caution, we nevertheless believe that these data provide some implications for how clinicians develop and provide support for siblings of children with ASD. When talking about their present attitudes, data indicate that the behaviour component of their attitude was most impacted by the presence of ASD, whereas when they were younger, it was the cognition component of attitudes that appeared most problematic. In other words, findings from this study indicate that support provided to young children should focus on providing an understanding of the disability and its potential impact on sibling relationships, as suggested by Glasberg (2000); while during adolescence, siblings may benefit most from support that focusses on improving their ability to modify their own behaviours during their interaction with their sibling with ASD.

Research has also demonstrated that siblings can have direct effects on one another’s development when they serve as social partners and role models as well as indirect influences through their impact on larger family dynamics – such as family structure, favouritism or diluting family resources (McHale, Updegraff & Whiteman, 2012). Therefore, speech-language therapists should capitalise on both these direct and indirect effects, for example, by supporting siblings in their communication partner role, as this has lifelong implications. This focus on siblings is also in line with American Speech-Language-Hearing Association (ASHA) guidelines on family-centred practice which underscores the role of siblings in speech-language intervention for contextualising intervention that reflects the family system and preferences, for paying attention to family priorities and concerns, for developing meaningful learning opportunities and teaching interaction skills to support and manage behaviour and the development of communication and language, and for recognising the transactional nature of a communication disorder (such as ASD) when evaluating family interactions and relationships (ASHA, 2017).

Certain methodological issues must be considered before generalising the findings too broadly. The first issue was the relatively small sample size that was recruited. Future research should endeavour to obtain a sample that is more representative of the sibling population within the South African context at large. The second issue was the self-report nature of the questionnaire, as participants may have provided responses that they thought were appropriate rather than truthful, thereby creating a Hawthorne effect. Self-report methodology is, however, the typical method of collecting data for attitudinal research, as no one can better report on their attitudes than the participants being surveyed. The third issue was the retrospective way that past attitudinal data were collected. Despite the fact that the LSRS (Riggio, 2000) has well-established reliability and validity in measuring the past and present state of sibling relationships, it was designed to be a recall measure. Therefore, a more accurate way to study changes in attitudes over time would be to use a longitudinal design. Using a longitudinal design would potentially provide a more accurate description of participants’ attitudes at different periods in time, as it does not rely on memories of experiences. This type of longitudinal design has its own disadvantages, particularly as far as participant attrition is concerned.

## Conclusion

The results of this study add to the empirical data documenting sibling relationships where one sibling has ASD. Findings indicate that the sibling attitudes change depending on whether the participant is thinking about their attitudes at the present moment, or when they recall their attitudes when they were younger. As a result, supports for siblings should be differentiated to account for the differences in the components of attitudes as reported in these data. Data suggest that sibling support interventions should acknowledge the developmental life stage of the sibling and focus on teaching strategies to bolster the component of attitude which is reported as being the least positive at that life stage. In conclusion, when designing and implementing supports for siblings of children with ASD, clinicians should use a lifespan approach to sibling attitudes to ensure that supports to bolster the cognition, affect and behaviour components which influence attitudes to the sibling relationship.
